# Cost-effectiveness analysis of fecal microbiota transplantation for inflammatory bowel disease

**DOI:** 10.18632/oncotarget.21491

**Published:** 2017-10-04

**Authors:** Ting Zhang, Jie Xiang, Bota Cui, Zhi He, Pan Li, Hai Chen, Lijuan Xu, Guozhong Ji, Yongzhan Nie, Kaichun Wu, Daiming Fan, Guangming Huang, Jianling Bai, Faming Zhang

**Affiliations:** ^1^ Medical Center for Digestive Diseases, The Second Affiliated Hospital of Nanjing Medical University, Nanjing 210011, China; ^2^ Center for Disease Control and Prevention, Wuxi 214000, China; ^3^ State Key Laboratory of Cancer Biology and Xijing Hospital of Digestive Diseases, The Fourth Military Medical University, Xi’an 710032, China; ^4^ Department of Biostatistics, School of Public Health, Nanjing Medical University, Nanjing 211100, China

**Keywords:** inflammatory bowel disease, fecal microbiota transplantation, cost-effectiveness analysis, incremental cost effectiveness ratio, net monetary benefit

## Abstract

There is a lack of health economics evidence on the use of fecal microbiota transplantation (FMT) for inflammatory bowel disease (IBD). This study aims to evaluate the cost-effectiveness before (with conventional therapy) and after introducing FMT for treating IBD. 104 patients with IBD received FMT were recruited. Health status was evaluated by European dimension health table (ED-5Q). Incremental cost-effectiveness ratio (ICER) and net monetary benefit (NB) were calculated by different age groups, genders, smoking status, and disease subtypes. The willingness-to-pay threshold was set to the value equal to three times China’s per capita GDP (141240 CNY/QALY, 2014). From the health-care perspective, FMT strategy was 73% likely to be cost-effective compared with the conventional therapy before FMT with an ICER of -185712 CNY/QALY and a positive NB of CNY 45150. From the societal perspective, FMT strategy was 75% likely to be cost-effective with an ICER of -207417 CNY/QALY and a positive NB of CNY 48395. Moreover, younger patients (≤ 24), females, non-smokers and Crohn’s disease (CD) achieved more benefits. This study for the first time demonstrated that FMT showed its cost-effectiveness, especially on improving the life quality and decreasing the medical and societal cost, for the moderate to severe IBD in a Chinese cohort.

## INTRODUCTION

Inflammatory bowel disease (IBD), comprising ulcerative colitis (UC) and Crohn’s disease (CD), is a chronic disease characterized by a relapsing and remitting course [1]. Genetics, immunology, gut microbial dysbiosis and environmental factors were involved in the pathogenesis of IBD [1, 2]. The incidence of IBD is increasing in Asia, from the results of a territory-wide IBD registry in Hong Kong, the adjusted prevalence of IBD, UC, CD, and IBD unclassified per 100,000 individuals in 2014 were 44.0, 24.5, 18.6, and 0.9, respectively [3].

Medical management of IBD involves the acute treatment for the induction of remission, followed by the maintenance of remission. Current treatment strategies for IBD consist of 5-aminosalicylic acid (5-ASA), corticosteroids, antibiotics, immunomodulators and biological agents, while some studies have shown that some probiotics, enteral nutrition and traditional Chinese medicine may also play an important role in maintaining long-term remission of IBD [4]. However, the huge financial burden and the limited clinical response are still primary challenges to patients. Researchers and physicians have never stopped to explore the novel therapies with cost-effectiveness for improving patients’ life quality.

Fecal microbiota transplantation (FMT), as a new strategy to treat gut dysbiosis, has showed its potential therapeutic role in IBD [5–16]. But the use of FMT in IBD is still controversial especially in Crohn's disease due to a lack of large randomized controlled trials to prove its efficacy. According to data (accessed by August 31, 2016) from www.clinicaltrials.gov, a total of 135 clinical trials on FMT have been registered, most of which were started from 2014 or afterwards. And most of the clinical trials focused on FMT for CDI or IBD. This trend further reflects the growing public interest in FMT for the treatment of IBD. Given new treatment strategies and drugs might bring a better clinical efficacy but with a larger economic burden to the patients, we should take the economic value of them into serious consideration. The high cost of IBD is driven especially by biological therapy and surgery, and FMT as an alternative therapy might be able to reduce the economic burden on patients and society. During our recent pilot studies [7, 10] for safety, feasibility, and efficacy of FMT on IBD, we recorded the medical and societal cost of patients during one year before and after FMT.

To ensure optimal distribution of treatment regimes for IBD, it is vital that new therapies should be evaluated in terms of medical economics and effectiveness. It is desirable to form medical policies based on the reliable evidence from the cost-effectiveness analysis. Therefore, this study aimed to evaluate the cost-effectiveness and economic value of FMT for the treatment of IBD in China.

## RESULTS

### Patient characteristics

A total of 222 patients with moderate to severe IBD underwent FMT in this study, and 118 patients were excluded according to the exclusion criteria. Then 104 patients were included in the final analysis (Table 1), consisting of 33 patients with UC and 71 patients with CD. The male-female ratio was 1.4:1 (61/43). The average age of patients was 34.4 ± 13.6 years old. The percentage of patients with a smoking history was 29.8% (31/104). The proportion of patients with a bachelor degree or above was 47.1% (49/104).

**Table 1:** Patient characteristics

Items	Results
Total number	104
Age (years), mean ± SD	34.4±13.6
Sex, male % (n)	58.7 (61)
Disease type	
UC % (n)	31.7 (33)
CD % (n)	68.3 (71)
Education background	
Beneath bachelor degree % (n)	52.9 (55)
Bachelor degree or above % (n)	47.1 (49)
Smoking, yes % (n)	29.8 (31)

UC: ulcerative colitis; CD: Crohn's disease; SD: standard deviation.

### Effectiveness

**Figure 1:**
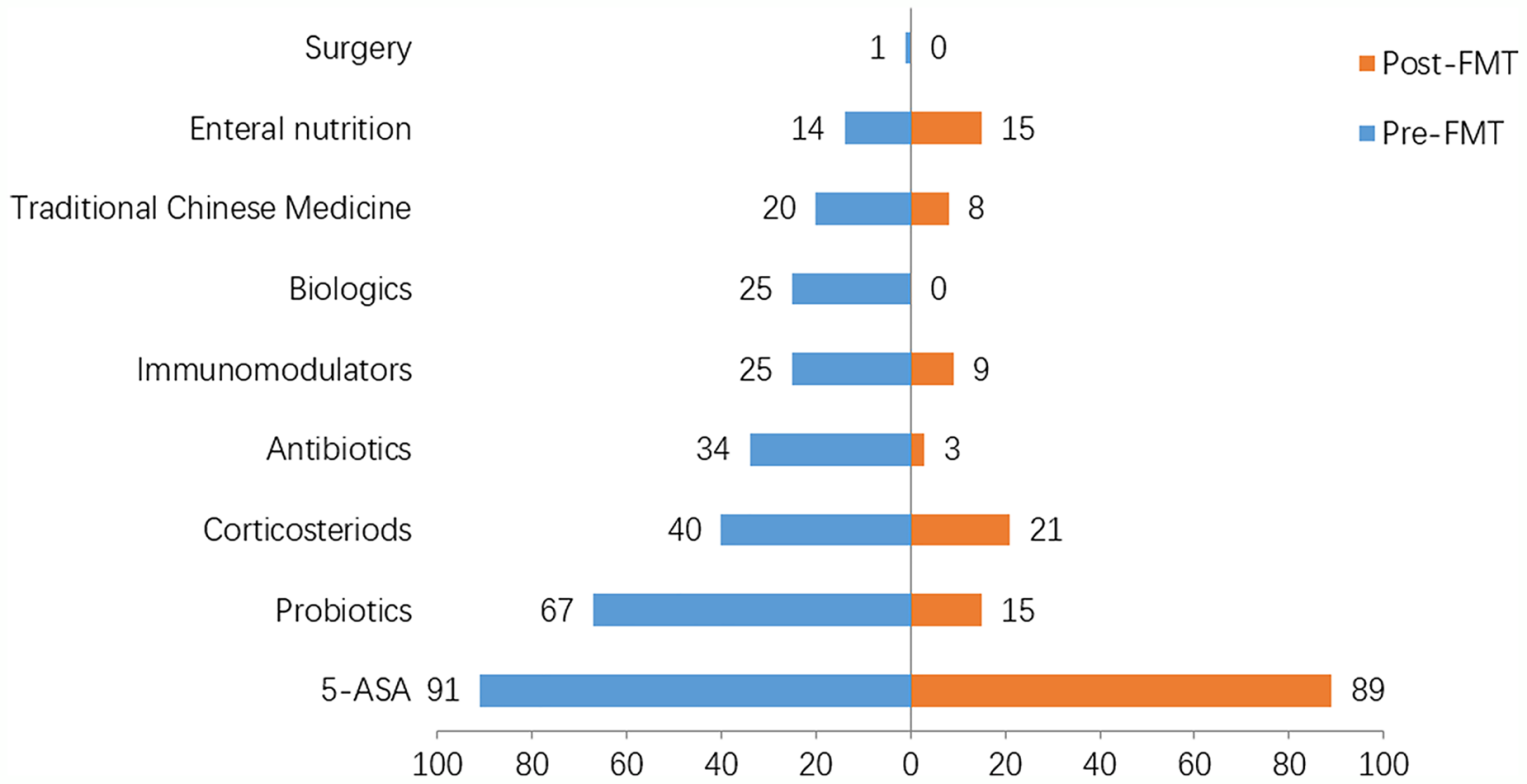
Comparison of treatment strategies between pre-FMT and post-FMT X-axis (abscissa): The proportion of people with the corresponding treatment. Y-axis (ordinate): The treatment strategies for patients.

In this study, we analyzed the changes on treatment strategies for IBD patients during one year before and after FMT. As shown in Figure 1, 5-ASA was the most important drug for maintaining clinical remission of IBD patients both before (87.5%, 91/104) and after FMT (85.6%, 89/104). The percentage of patients receiving the treatment of corticosteroids pre-FMT was 38.5% (40/104), but it decreased by nearly half post-FMT 20.2% (21/104). Besides, the rate of patients who used biologics decreased from 24.0% (25/104) before FMT to 0% after FMT. The utilization ratio of antibiotics, immunomodulators and Chinese traditional medicine also decreased after the treatment of FMT. The proportion of patients receiving the treatment of enteral nutrition remained relatively steady during one year before and after FMT.

### Cost analysis

As is shown in Table 2, the average physician office visits between one year pre-FMT and one year post-FMT were 8.2 and 6.3 (*p*=0.051). After the implementation of FMT therapy, the average hospitalization days showed a significant reduction (20.6 *vs.* 52.5, *p*=0.048). Average total medical costs between one year before and after FMT were CNY 77197.7 and CNY 51383.9, reflecting a significant drop with the treatment of FMT (*p*=0.030). Loss of working time before and after FMT was 85.4 days and 34.9 days (*p*=0.022). The average societal cost before and after FMT was CNY 89552.0 and CNY 60722.0 (*p*=0.020).

**Table 2:** Comparison of cost items between pre-FMT and post-FMT

Items	Pre-FMT	Post-FMT	*P*
Physician office visit (times), mean ± SD	8.2±2.3	6.3±1.7	0.051
Hospitalization (days), mean ± SD	52.5±18.5	20.6±11.2	0.048
Loss of working time (days), mean ± SD	85.4±13.5	34.9±10.8	0.022
Total medical costs (CNY), mean ± SD	77197.7±10385.2	51383.9±8553.6	0.030
Costs for loss of work (CNY), mean ± SD	9375.4±2230.7	7130±862.8	0.049
Societal costs (CNY), mean ± SD	89552.0±15035.8	60722.0±14950.6	0.020

FMT: fecal microbiota transplantation; CNY: China Yuan; SD: standard deviation.

### Cost-effectiveness analysis

**Figure 2:**
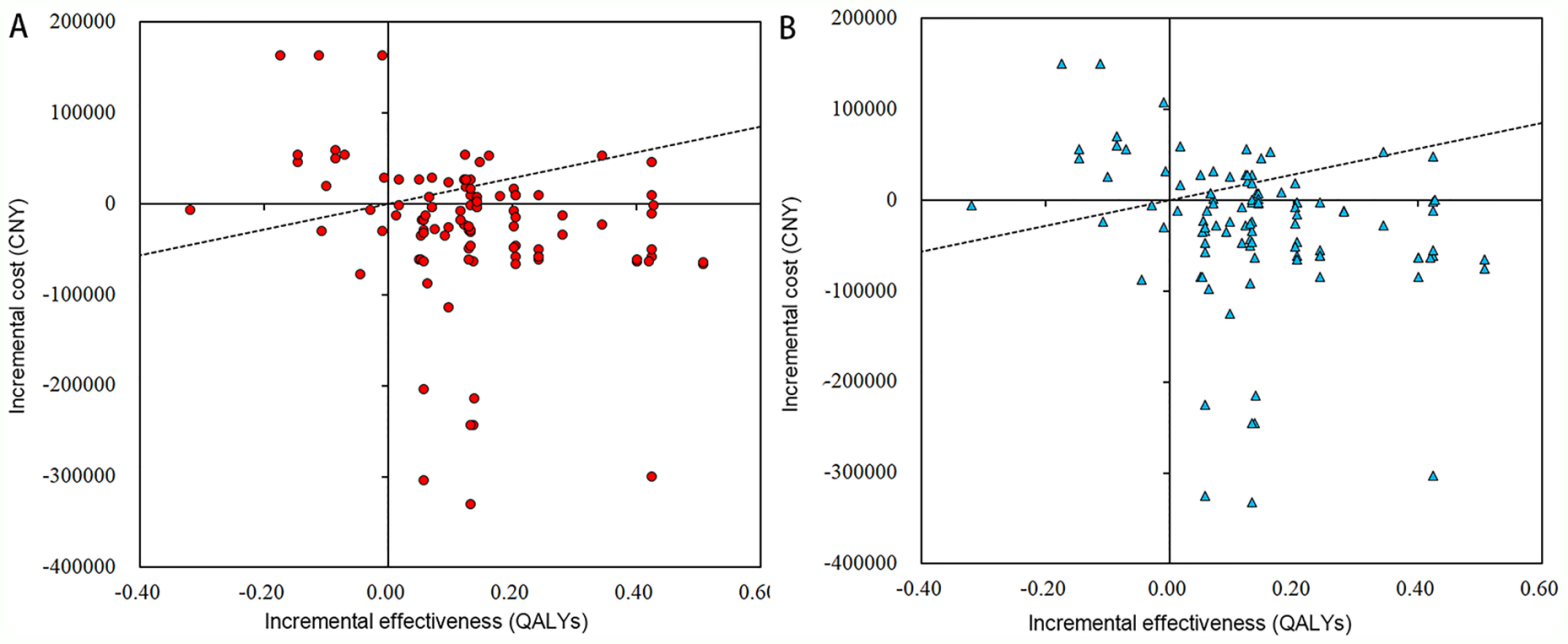
Cost-effectiveness plane on the medical and societal perspectives **(A)** The total medical cost decreased and the total health effect increased after FMT within one year, indicating that FMT was cost-effective on the medical perspective. **(B)** The total societal cost decreased and the total health effect increased after FMT within one year, indicating that FMT was cost-effective on the societal perspective.

The analysis for the cost-effectiveness of FMT was shown in Table 3. Based on the willingness-to-pay threshold of 141240 CNY/QALY, 2014, from the medical perspective, FMT was associated with an overall increase in QALYs of 0.139 and an increase in cost of CNY 25814 compared with that before FMT. It suggested that FMT was a cost-effective option for IBD patients with an ICER of -185712 CNY/QALY and a positive NB of CNY 45150. From the societal perspective, FMT was associated with an overall increase in QALYs of 0.139 and an increase in cost of CNY 48395 compared with that before FMT. Similarly, it also indicated that FMT was a cost-effective option for IBD patients with an ICER of -207417 CNY/QALY and a positive NB of CNY 48395. The cost-effectiveness plane for FMT was presented in Figure 2.

**Table 3:** Cost-effectiveness analysis on the medical and societal perspectives

Perspective	Time	Total effectiveness (QALYs)	Total cost (CNY)	Incremental effectiveness QALYs)	Incremental cost (CNY)	NB (CNY)	ICER (CNY/QALY)	<1×per capita GDP	<3×per capita GDP
Medical	Pre-FMT	0.634	77198						
	Post-FMT	0.773	51384	0.139	-25814	45150	-185712	√	√
Societal	Pre-FMT	0.634	89552						
	Post-FMT	0.773	60722	0.139	-28831	48395	-207417	√	√

FMT: fecal microbiota transplantation; QALY: quality adjusted life years; NB: net monetary benefit; CNY: China Yuan; ICER: incremental cost effectiveness ratio.

### Subgroup analysis

According to Table 4, the baseline factors such as the patients’ age, gender, smoking status and disease type were included for assessing the cost-effectiveness of FMT. In subgroup analysis towards different baseline factors, FMT remained cost-effective with an improved health benefit and a reduced cost. As for the different age groups, patients who were less than or equal to 24 year-old benefited most with a positive NB of > CNY 60000. As for the disease classification, patients with CD achieved more health and economic benefits with a positive NB of > CNY 50000. And from both medical and societal perspectives, female patients achieved more cost-effectiveness, as well as the non-smokers did.

**Table 4:** Cost-effectiveness analysis on common risk factors related to inflammatory bowel disease

Items	Classification	Perspective	Incremental effectiveness (QALYs)	Incremental cost (CNY)	NB (CNY)	ICER (CNY/QALY)	<1×per capita GDP	<3×per capita GDP
Age (years)	≤24	Medical	0.108	-45826	61080	-424314	√	√
		Societal	0.108	-50115	65369	-464028	√	√
	24-33	Medical	0.134	-33581	52507	-250604	√	√
		Societal	0.134	-36754	55680	-274284	√	√
	33-43	Medical	0.147	-4508	25270	-30667	√	√
		Societal	0.147	-6104	26866	-41524	√	√
	>43	Medical	0.170	-18056	42067	-106212	√	√
		Societal	0.170	-20861	44872	-122712	√	√
Sex	male	Medical	0.125	-23243	40898	-185944	√	√
		Societal	0.125	-26043	43698	-208344	√	√
	female	Medical	0.158	-30692	53008	-194253	√	√
		Societal	0.158	-33985	56301	-215095	√	√
Smoking	yes	Medical	0.129	-25912	44132	-200868	√	√
		Societal	0.129	-28995	47215	-224767	√	√
	no	Medical	0.143	-26432	46629	-184839	√	√
		Societal	0.143	-29399	49596	-205587	√	√
Disease type	UC	Medical	0.142	-15473	35529	-108964	√	√
		Societal	0.142	-18498	38554	-130267	√	√
	CD	Medical	0.137	-31152	50502	-227387	√	√
		Societal	0.137	-34142	53492	-249212	√	√

QALY: quality adjusted life years; NB: net monetary benefit; CNY: China Yuan; ICER: incremental cost-effectiveness ratio; UC: ulcerative colitis; CD: Crohn's disease.

### Sensitivity analysis

**Figure 3:**
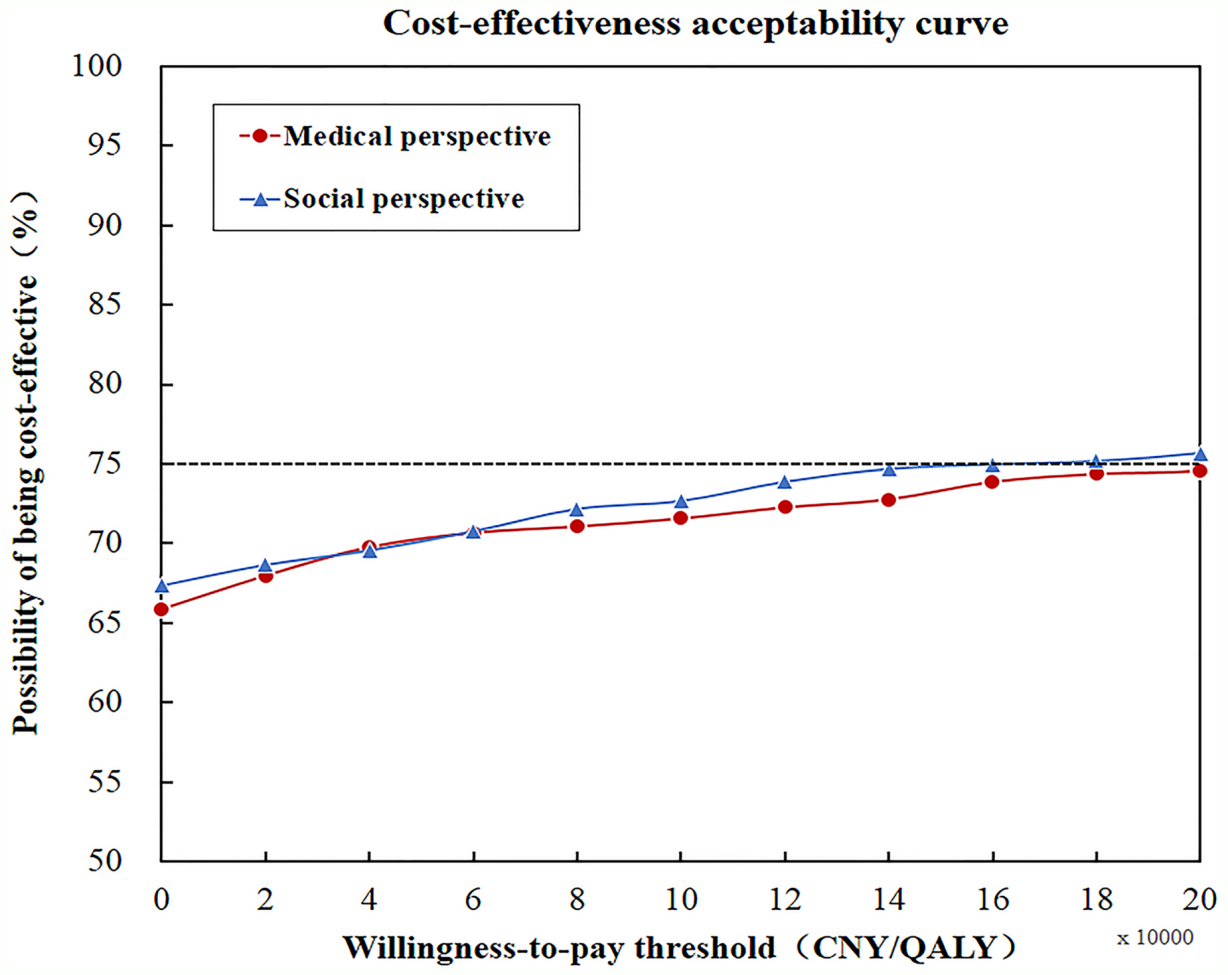
Sensitivity analysis by cost-effectiveness acceptability curve On the premise that willingness-to-pay threshold was defined as 141240 CNY / QALY, potential possibility of FMT strategy being cost-effective was 0.73 (medical perspective) and 0.75 (societal perspective), which showed that FMT was totally cost-effective.

Probabilistic sensitivity analysis, varying all parameters simultaneously, showed that the results were robust with the majority of Monte Carlo simulations clustered together. As shown in the CEAC (Figure 3), on the premise that the willingness-to-pay threshold be defined as 141240 CNY / QALY, the potential possibility of FMT being cost-effective was 0.73 (medical perspective) and 0.75 (societal perspective).

## DISCUSSION

There were few studies focusing on the cost-effectiveness analysis of FMT for CDI or other treatments for IBD, but there was no cost-effectiveness report on FMT for IBD. Varier et al. [17] reported the cost-effectiveness analysis of FMT versus vancomycin in the treatment of recurrent CDI in 2015, which showed that FMT was more cost-effective and could reduce the financial burden on patients. Blackhouse et al. [18] reported that infliximab (IFX) for refractory CD was not more cost-effective compared with the conventional treatment. Our study first time evaluated the cost-effectiveness of FMT for the treatment of IBD.

Our aim was to evaluate that whether the treatment with FMT for moderate to severe IBD was superior to previous traditional therapies from medical economic perspectives quantified by cost-effectiveness analysis. We found that the use of some drug (i.e., steroids and IFX) was reduced or stopped after FMT. While health outcomes improved overall, the medical costs correspondingly reduced. Moreover, this study showed that switching from previous medication therapy to FMT could effectively improve the patients’ life quality and relieve the patients’ economic burden.

In this study, as for the medical treatments for IBD, 5-ASA was used as the dominated maintenance drug both during one year before and after FMT. This observation consisted with the report that 5-ASA is currently the first-line drug for maintaining clinical remission in patients with mild to moderate IBD. Instead, the use of other drugs, with particular to the biological agents and corticosteroids, were significantly reduced post FMT in this study. In recent years, biological agents represented by IFX have made a significant progress in the treatment of IBD, especially for the patients with moderate to severe IBD who are refractory to the traditional therapy. However, some IBD patients achieved no response to IFX, and the incidence rate of primary non-response was about 10% to 30% [19]. The secondary non-response rate was 23% to 46%, which was defined as re-emergence of symptoms in IBD patients who had achieved clinical remission from the initial treatment [20].

In our study, the IFX utilization ratio before FMT was 24% because IFX was not covered by medical insurance in China in recent years. These patients then underwent FMT and remained free of IFX within one year after the treatment. More importantly, they achieved a significant health benefit post-FMT. Taking the adverse effects of IFX into consideration, including increased opportunistic infections, infusion reactions and high medical costs, FMT might have a better cost-effectiveness for the patients who had no response to IFX. The cost-effectiveness of the two treatment modalities will be further compared horizontally in our ongoing multi-center clinical trial.

In the present study, another significant change in the treatment strategy after FMT was an obvious reduction in the use of steroids. Steroid is highly effective and can rapidly induce clinical remission, making it a popular choice for patients with moderate to severe active UC. However, once the dosage of steroids is reduced, the patient's clinical symptoms may relapse. This is defined as steroid-dependent ulcerative colitis (SD-UC). Considering the long-term use of steroids might bring additional risks to patients, it remains a challenge on controlling the symptoms of UC effectively without the use of steroids. In our study, 38.5% of patients used steroids pre-FMT, but the rate decreased by nearly half post-FMT. This might provide a new treatment approach of FMT for treating SD-UC. Combining with conventional treatment strategies, FMT might help patients effectively control their disease symptoms and get away from the use of steroids. Collectively, for IBD patients, the step-up FMT strategy involving medicine after FMT was proved to be more effective than that before FMT [10].

In this study, the stratification factors, including the age, the gender, the smoking status and the disease type, were analyzed to assess which group could benefit more. As shown in Table 3, patients who were less than or equal to 24 year-old benefited most. Patients with CD were observed to achieve more clinical and economic benefits. In addition, the non-smokers and the females could obtain a better clinical efficacy at a less cost. It suggested that an appropriate patient population should be preferred to undergo FMT for achieving more health and economic benefits.

More importantly, this study evaluated the cost-effectiveness of FMT for patients with IBD. In terms of the effectiveness, the utility value of life quality increased 0.14 after FMT. In terms of the cost, the advantages of FMT derived mainly from the savings on medical expenses after FMT. From the medical and societal perspective, ICER of FMT was -185712 CNY/QALY and -207417 CNY/QALY, respectively. It showed that FMT strategy was totally cost-effective in treating IBD patients on both medical and societal perspectives. Collectively, FMT is a useful strategy, which could not only improve health benefits but also reduce costs.

However, there are several limitations in this study. First, this study is a non-controlled randomized trial carried out at our single medical center, making the results might not fully reflect the actual state of treatment for IBD in our country. A larger sample study in multiple centers from Chinese or other populations should be performed to verify the data. Moving forward, even for the economic assessment for a medical technology, we should ideally adopt a randomized controlled design to reduce the effects from potential biases. The second limitation is that the overall calculated costs were partly based on the estimates, which used some variables like GDP per capita to predict the specific societal costs. It may not truly represent the actual medical and social costs of patients.

Another limitation is that it is a pre-post study, which has several disadvantages. As previous study reported, the disadvantages include regression to the mean, time period bias, lack of a control group, and asymmetrical treatment durations [21]. In this trial, a treatment regime was performed where the effectiveness and the cost were compared about 12 months before and after switching to FMT therapy for patients with IBD. Therefore, some changes in the factors such as the medical payment revisions and the patient’s age, however small, should be considered to reduce the influence on the results. Although these discussion points have been investigated in the sensitivity analysis, it is possible that this may not be sufficient. These factors should be further analyzed in future studies.

## MATERIALS AND METHODS

### Recruitment of patients

A prospective study on patients with IBD as a part of clinical trial (NCT01790061, NCT01793831) was carried out by the Medical Center for Digestive Diseases at the Second Affiliated Hospital of Nanjing Medical University, Nanjing, China, from October 2012 to December 2015. This study was reviewed and approved by the Second Affiliated Hospital of Nanjing Medical University Institutional Review Board. All eligible subjects provided written informed consents prior to participation in this study. The diagnosis of IBD was made according to the clinical, endoscopic, and histological criteria [22]. Disease severity was evaluated by Mayo score for UC and Harvey Bradshaw Index (HBI) for CD. All included patients with moderate to severe IBD underwent FMT and were followed up for more than one year. Exclusion criteria included subjects who had mild IBD, subjects with disease duration of less than one year; subjects who had an active acute or chronic infectious disease; subjects with a clinically significant disease e.g. cancer, myocardial infarction, cerebrovascular disease and stroke; and subjects with incomplete samples or data.

### Preparation of fecal microbiota and FMT procedure

Healthy donors were screened with a rigorous set of criteria according to our previously published report [7]. For the laboratory procedure, the fresh stool from donors were originally prepared with the method of Filtration plus Centrifugation (FPC) [7]. Subsequently a new method termed microfiltration plus centrifugation (MPC), which was based on an automatic microbiota purification system named GenFMTer (FMT Medical, Nanjing, China) [10], was used for the purification of fresh fecal microbiota. We controlled the process time from defecation to the fresh bacterial material be infused into the patient's intestines within one hour based on the automatic purification system GenFMTer and the corresponding laboratory and clinical pathway [10]. This method was defined as “one-hour FMT protocol” [16]. The prepared fecal microbiota suspension was injected into the distal duodenum of recipients through gastroscope under anesthesia. One hour before FMT, patients were given metoclopramide 10 mg by intramuscular injection and esomeprazole magnesium 40 mg intravenously to prevent the microbiota liquid reflux and inhibit the secretion of gastric acid. Another delivering way for FMT was through colonic transendoscopic enteral tubing (TET) [23], which is a new endoscopic interventional technology for the whole colon administration of fresh microbiota liquid suspension.

### Outcomes

The clinical outcome measure of this study was the QALY evaluated by the EuroQol-5 dimensions (EQ-5D) [24]. The patient’s quality of life was assessed by Standard EQ-5D at the baseline pre-FMT and one year post-FMT. The EQ-5D questionnaire includes five dimensions: mobility, self-care, usual activities, pain or discomfort, anxiety or depression. Each dimension contains three levels: having no problems, having some or moderate problems, being unable to do/having extreme problems. The health status of the five dimensions can be converted into a preference weight as the EQ-5D index score through the Chinese time trade-off (TTQ) value table [25] in this study. Complete health was defined as “1” and death was “0”. As for statistical analysis, the EQ-5D health utility value was used as a continuous variable and the changes from the baseline before FMT to one year after FMT was analyzed by a paired t-test.

### Cost

The data on cost was obtained from billing details according to the hospital information system and the patients’ own billing records. The total costs consisted of medical costs and non-medical costs. Medical costs included hospitalization costs and outpatient costs. Drug costs during hospitalization, inspection costs, operating costs, bed fees and care costs were included in the hospitalization costs, whereas registration fees, diagnosis costs, examination fees and outpatient drug costs were included in the outpatient costs. Non-medical costs were derived from a loss of working time in patients and their family members.

### Cost-effectiveness analysis

In this study, the primary outcome measure was incremental cost-effectiveness ratio (ICER) and net monetary benefit (NB), which were calculated based on the cumulative costs and cumulative effects during one year before and after FMT.

$$ICER=\frac{C_{1}-C_{2}}{E_{1}-E_{2}}=\frac{\Delta C}{\Delta E}$$

$$NB=\lambda \times \left(E_{1}-E_{2}\right)-\left(C_{1}-C_{2}\right)=\lambda \times \Delta E-\Delta C$$

C_1_ is the total cost of comparative regimen (higher), C_2_ is the total cost of reference treatment regimen, E_1_ is the total effectiveness of comparative regimen (higher), E_2_ is the total effectiveness of reference regimen and λ is the cost-effective threshold (willingness-to-pay). In this study, three times of GDP per capita in China worked as the cost-effective threshold.

FMT as an interventional treatment was evaluated according to the international recommendations of the World Health Organization: ICER < per capita GDP (be highly cost-effective); per capita GDP < ICER < 3 times per capita GDP (be cost-effective); ICER > 3 times the per capita GDP (not cost-effective). The willingness-to-pay threshold was set to the value equal to three times China’s per capita GDP (141240 CNY/QALY, 2014). At the same time, to evaluate the influence of baseline factors such as age, gender, smoking status, and disease type on the cost-effectiveness of FMT, subgroup analysis was carried out to assess which group could benefit more.

A comparison of the cost and health outcomes of the two regimens between one year pre-FMT and post-FMT can be expressed by the four quadrants in the cost-effectiveness plane. The abscissa represents the incremental health outcomes post-FMT compared with that pre-FMT, and the ordinate represents the incremental cost post-FMT compared with that pre-FMT. When the results fell within the quadrant II / IV, the cost increased while the effect decreased (II), or the cost decreased while the effect increased (IV), which indicating that one group has absolute disadvantage / advantage compared with the other group. At this point, a conclusion of rejection / acceptance for the new strategy can be drawn directly without further cost-effective analysis.

### Sensitivity analysis

Probabilistic sensitivity analysis was used to explore the robustness of the results. The cost parameters and the EQ-5D index scores were changed at the same time to assess the stability. The cost parameter is set to Gamma distribution and the utility value parameter is set to Beta distribution. The cost-effectiveness plane shows the results of the probabilistic sensitivity analysis, including a series of points from the second order Monte Carlo simulation (1000 iterations). Based on the cost-effectiveness plane, cost-effectiveness acceptability curve (CEAC) was drawn.

## CONCLUSION

In conclusion, this study for the first time demonstrated that FMT showed its cost-effectiveness, especially on improving the life quality and decreasing the medical and societal cost, for the moderate to severe IBD in a Chinese cohort. To maximize health and economic benefits, the appropriate patient population should be recommended to receive FMT according to the different baseline factors.

## Abbreviations

FMT: fecal microbiota transplantation
IBD: inflammatory bowel disease
UC: ulcerative colitis
CD: Crohn's disease
IFX: infliximab
(5-ASA): 5-aminosalicylic acid
HBI: Harvey-Bradshow Index
QALY: quality adjusted life years
NB: net monetary benefit
CNY: China Yuan
ICER: incremental cost effectiveness ratio
